# *Grandidierella gilesi* Chilton, 1921 (Amphipoda, Aoridae), first encounter of non-indigenous amphipod in the Lam Ta Khong River, Nakhon Ratchasima Province, North-eastern Thailand

**DOI:** 10.3897/BDJ.8.e46452

**Published:** 2020-03-06

**Authors:** Koraon Wongkamhaeng, Pongrat Dumrongrojwattana, Myung-Hwa Shin, Chaichat Boonyanusith

**Affiliations:** 1 Kasetsart University, Bangkok, Thailand Kasetsart University Bangkok Thailand; 2 Burapha University, Bangsaen, Thailand Burapha University Bangsaen Thailand; 3 National Marine Biodiversity Institute of Korea, Seocheon, South Korea National Marine Biodiversity Institute of Korea Seocheon South Korea; 4 Nakhon Ratchasima Rajabhat University, Nakhon Ratchasima, Thailand Nakhon Ratchasima Rajabhat University Nakhon Ratchasima Thailand

**Keywords:** Aorid amphipod, Crustacea, non-indigenous, aquaculture, Lam Ta Khong River, Thailand

## Abstract

The first record of the non-indigenous, alien amphipod *Grandidierella gilesi* in the Lam Ta Khong River is presented. Previously, this Indo-Pacific amphipod had only been reported in the Indian Ocean, the Andaman Sea, the Gulf of Thailand, the South China Sea and Australia. In Thailand, *G. gilesi* was previously reported in an isolated pond in Bangkok. The present study constitutes another record of this species in inland water. The characteristics and variation of *G. gilesi*, observed in this study, are also discussed. All the specimens described here are preserved at the Princess Maha Chakri Sirindhorn Natural History Museum, Prince of Songkla University, Songkla, Thailand.

## Introduction

Forty-three valid species of *Grandidierella* Coutière, 1904 have been recorded around the world (Horton et al. 2019). The members of this genus have circumtropical distribution. Two species of *Grandidierella*, *G. bonnieroides* Stephensen, 1947 and *G. japonica* Stephensen, 1938, are known as widespread non-indigenous amphipods. *Grandidierella bonnieroides* typically occurs around tropical areas, including the Saudi Coast, the Red Sea and the Suez Canal (Myers 1981). This species was recorded in the Mediterranean Sea in a 2016 study, in which the author suggested that it may have travelled by ship transportation through the Suez Canal (Lo Brutto et al. 2016). *Grandidierella japonica* was originally described in Japan and introduced to the entire Pacific Ocean via the oyster trade and ship transportation in the 1960s (Chapman and Dorman 1975). It has invaded a high number of estuaries in Pacific North-America (Pilgrim et al. 2013) and has been spotted from several sites in Atlantic Europe. Recently, the arrival of *G. japonica* into the Mediterranean Sea has been recorded and further spreading is expected (Marchini et al. 2016).

**Figure 1.**
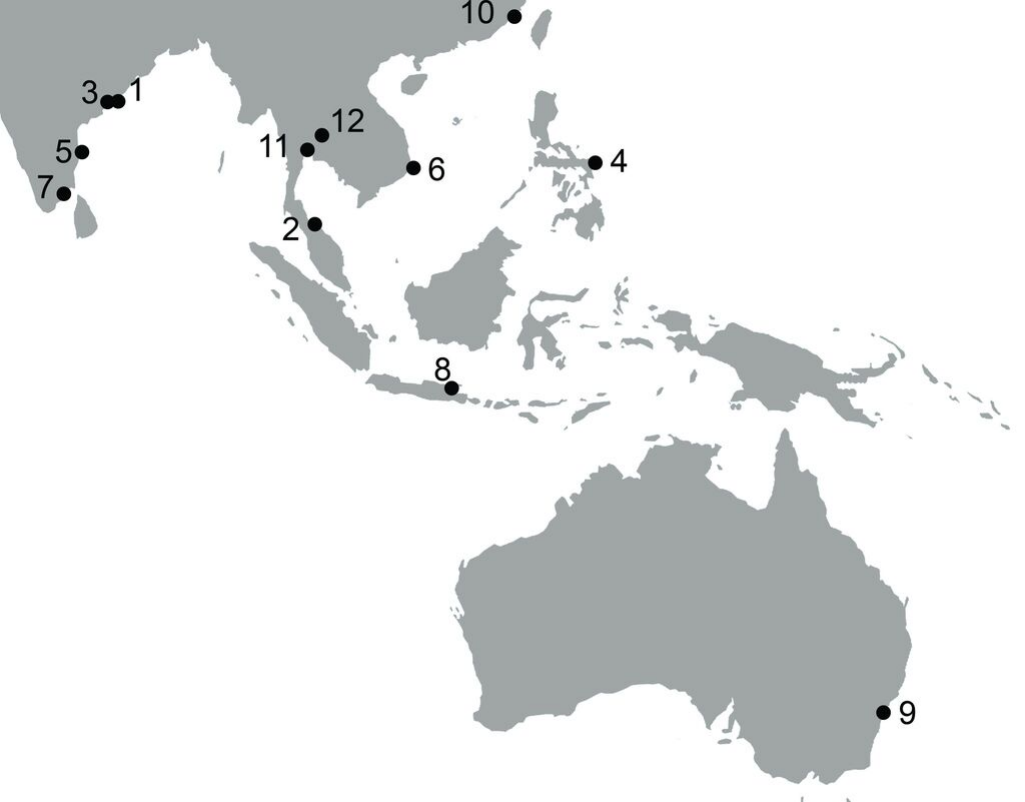
Distribution of *Grandidierella gilesi*: 1. Chilika Lagoon, India (Chilton 1921); 2. Songkhla Lake (Tale Sap) and Pattani River and Malaysian State (Chilton 1925); 3. large creek close to Tumidalametta Hill at 102 m elev. in Vizagapatam, India (KHBarnard 1935); 4. Lapinig, Philippines (Schellenberg 1938); 5. Adyar, Madras Coast, India (Nayar 1959); 6. Nhatrang, Vietnam (Imbach 1967); 7. Pinnakayal, India (Sivaprakasam 1970); 8. Surabaya, Java, Indonesia (Ledoyer 1979); 9. Port Beacon, Brooklyn Gut, Hawksbury River, New South Wales (Myers 1981); 10. Qi'ao-Dan'gan Island Mangrove Nature Reserve on Qi'ao Island in the Pearl River Estuary, China (Wang et al. 2010); 11. Man-made freshwater pond in Kasetsart University, Bangkok, Thailand (Wongkamhaeng et al. 2016) and 12. Lam Tha Kong River, Thailand (present study).

*Grandidierella gilesi* Chilton, 1921 is a euryhaline species that has been reported in various habitats in the Indo-Pacific area (Fig. 1). The species generally occurs in polluted brackish waters, in which it constructs tubes on various types of hard substrate in association with *Jassa* sp. and polychaetes (Barnard et al. 1991). This species was first described in brackish water in Chilika Lake, India in 1921 (Chilton 1921). Later, *G. gilesi* was recorded in a large creek close to Tumidalametta Hill at 102 m elev. in Vizagapatam, India (Barnard 1935); this shows that the amphipod had migrated from the sea and settled in a fresh water habitat. *Grandidierella gilesi* has been recorded in other estuarine places, such as in Songkhla Lagoon (recorded as Tale Sap), the Pattani River (Chilton 1925) and Lapinig in the Philippines (Schellenberg 1938). It has also been reported in marine waters, such as in the Madras Coast, India (Asari and Myers 1982, Nayar 1959); the Natrang Coast, Vietnam (Imbach 1967); the Pinnakayal Coast, India (Sivaprakasam 1970); Surabaya, Java, Indonesia (Ledoyer 1979) and in the Port Beacon, Australia (Myers 1981).

The Lam Ta Khong River is a major river in Nakhon Ratchasima Province, originating from the Dong Phaya Yen Ranges (Khao Yai National Park). It flows eastwards through the Pak Chong, Sikhiu, Sung Noen and Mueang Nakhon Ratchasima Districts over a distance of 224 km. The Lam Ta Khong Dam was constructed at the border of the Pak Chong and Sikhiu sub-district (Tesana 2002). The Lam Ta Khong River is part of the Mun River Basin, which is a tributary of the Mekong River. The Lam Ta Khong River is an important river in terms of agriculture and pumped storage. The river suffers from anthropogenic effects, including sewage and pollution (Setpong and Chuersuwan 2015). *Grandidierella gilesi* was found in a benthic macroinvertebrate sampling of the Lam Ta Khong River in the Sung Noen District.

**Figure 2.**
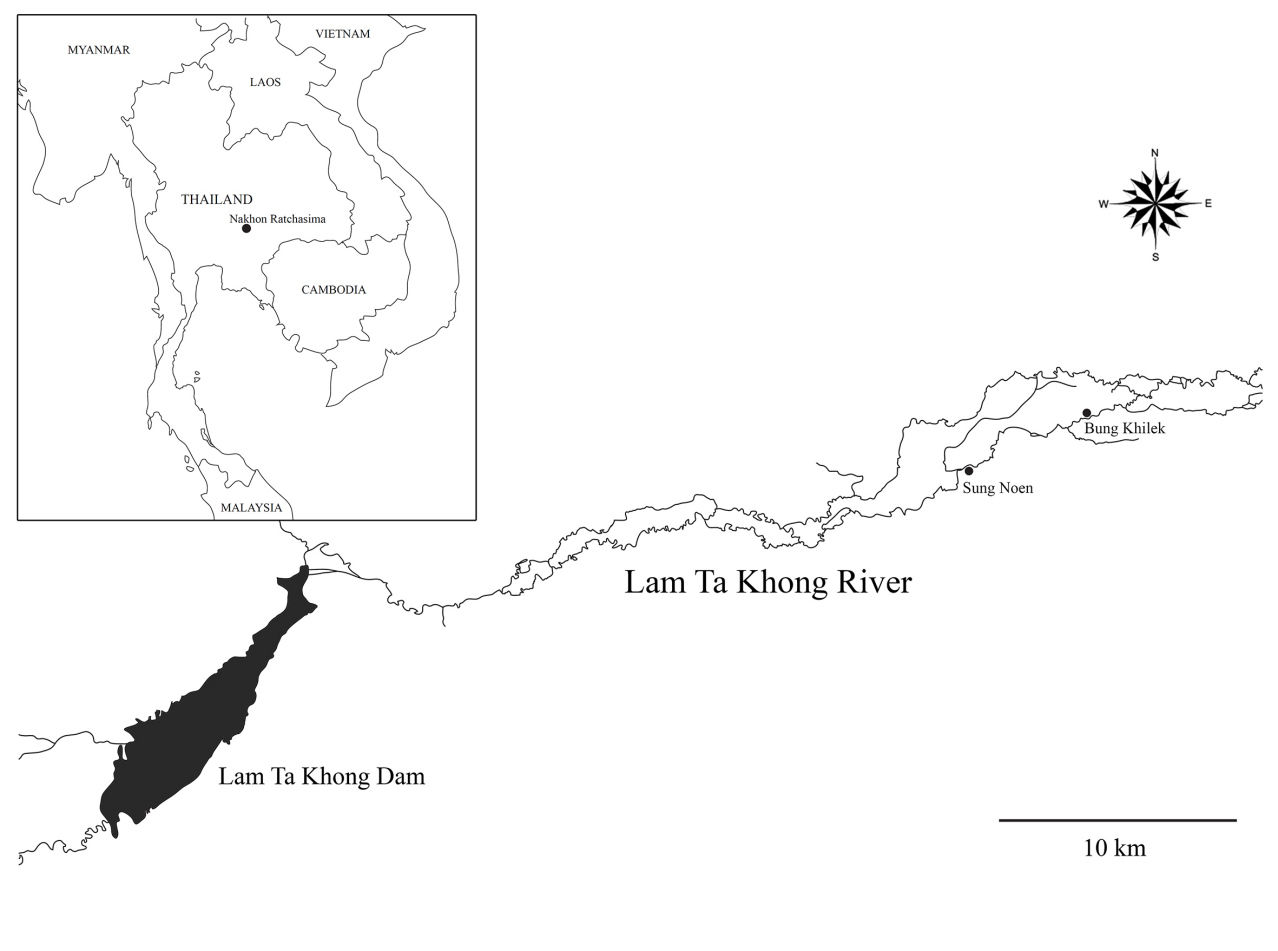
Study site in the Lam Ta Khong River, Nakhon Ratchasima Province, Thailand

The Lam Ta Khong River is situated in the northeast part of Thailand (Fig. 2). This river is divided into upper and lower areas by the Lam Ta Khong Dam. The present study was conducted on the lower area of the Lam Ta Khong River, covering an area of 2,210 km^2^. Over the length of 120 km, this river receives pollution loads from anthropogenic activities including municipal, agricultural and industrial wastewaters. The Lam Ta Khong River Basin is influenced by the Southwest and Northwest monsoons, with annual rainfall around 1,000 mm (Setpong and Chuersuwan 2015). This area consists of farmland, forest, residential areas and aquacultural areas.

Amphipods were collected in the Lam Ta Khong River at Sung Noen (14°54'06.6"N, 101°48'53.7"E), which has a silty clay bottom and at Bung Khilek (14°55'12.5"N, 101°51'43.2"E), which has a loamy sand bottom. The depth of both areas were 1.5 m and no aquatic plants were identified. The water temperature ranged from 20–34°C. The samples were collected in July and November 2016 using a D-frame net and then fixed in 10% buffered formalin. Amphipod specimens were then sorted out and stored in 70% alcohol. Twenty specimens were examined under a stereomicroscope and dissected. A photograph (Canon EOS) was taken of each specimen and the total length of each specimen was measured from the tip of the rostrum to the apex of the telson using Acrobat Pro X. Drawings were produced using a drawing tube attached to an Olympus CH30 light microscope. These pencil drawings were scanned and digitally inked using a WACOM bamboo CTH-970 graphics board, according to the method described by Coleman (2003). Specimens were deposited in the Prince of Songkla University Zoological Collection (PSUZC). The following abbreviations are used in the figures: A, antenna; Gn, gnathopod; LL, lower lip; Md, mandible; Mx, maxilla; Mp, maxilliped; P, pereopod; PL, pleopod; T, telson; UL, upper lip; Ur, uropod.

## General information

## Habitat

The amphipod is a euryhaline species and has been recorded in soft bottom habitats from open seas to estuaries with salinities ranging from 15 ppt to 32 ppt. This amphipod is a tube builder and it attaches its tubes to hard substrates, including sticks from trees, algae and oyster shells.

Soft bottom, brackish water with salinity range from 15 ppt to 32 ppt.

12. Marine Intertidal

15. Artificial/Marine

## Distribution

Chilika Lagoon, India (Chilton 1921); Songkhla Lake (Tale Sap) and Pattani River and Malaysian State (Chilton 1925); large creek close to Tumidalametta Hill at 102 m elev. in Vizagapatam, India (Barnard 1935); Lapinig, Philippines (Schellenberg 1938); Adyar, Madras Coast, India (Nayar 1959); Nhatrang, Vietnam (Imbach 1967); Pinnakayal, India (Sivaprakasam 1970i); Surabaya, Java, Indonesia (Ledoyer 1979); Port beacon, Brooklyn Gut, Hawksbury River, New South Wales (Myers 1981);

Sung Noen, Lam Ta Khong River 14°54'06.6"N, 101°48'53.7"E, silty clay, depth 1.5 m and Bung Khilek, Lam Ta Khong River, 14°55'12.5"N, 101°51'43.2"E, loamy sand bottom, depth 1.5 m, 200 km from the Gulf of Thailand. According to data from the Department of Fisheries, pathways of introduction may possibly be related to the *Litopenaeus vannamei* farms in the abandoned saltpan in None Pradu District, 50 km from the study site. In 2011, there was a massive flood, covering two-thirds of the country which included this area. The amphipod, living in the aquaculture system, may have migrated to the Lam Ta Khong River during the flood at that time.

## Newly reported occurrences

- **Type status:** Other material. **Occurrence:** occurrenceDetails: Asari KP, Myers AA (1982); catalogNumber: PSUZC- 0449; occurrenceRemarks: silty clay, depth 1.5 m, 0 ppt; recordNumber: Boonyanusith, C., D-frame net collecting,; recordedBy: Koraon Wongkamhaeng; individualID: Grandidierella gilesi; individualCount: 16; sex: male; lifeStage: adult; reproductiveCondition: mature; behavior: tube builder, attached to a log at the bottom of the river; occurrenceStatus: Dominant, lives with aquatic insects; preparations: 70% alcohol; disposition: deposited at Princess Maha Chakri Sirindhorn Natural History Museum, Prince of Songkla University, Songkla, Thailand; occurrenceID: urn:catalog:PSUZC:Crus:0449; **Taxon:** taxonID: Introduced; taxonConceptID: Koraon Wongkamhaeng; scientificName: Grandidierella gilesi; kingdom: Animalia; phylum: Arthropoda; class: Malacostraca; order: Amphipoda; family: Aoridae; genus: Grandidierella ; specificEpithet: gilesi; scientificNameAuthorship: Chilton, 1921; **Location:** locationID: 14°54'06.6"N, 101°48'53.7"E; continent: Asia; waterBody: river; country: Thailand; stateProvince: Nakhon Ratchasima; county: Thailand; municipality: Sung Noen; locality: Lam Ta Khong River; verbatimElevation: 212 m; verbatimDepth: 1.5 m; verbatimCoordinates: 14°54'06.6"N, 101°48'53.7"E; georeferenceProtocol: GPS; **Identification:** identifiedBy: Koraon Wongkamhaeng; dateIdentified: 2016; **Event:** samplingProtocol: D-frame net collecting; eventDate: 07/07/2016; **Record Level:** modified: 09/05/2019; language: en; collectionCode: Crustacea; basisOfRecord: PreservedSpecimen
- **Type status:** Other material. **Occurrence:** occurrenceDetails: Asari KP, Myers AA (1982); catalogNumber: PSUZC- 0449; occurrenceRemarks: silty clay, depth 1.5 m, 0 ppt; recordNumber: Boonyanusith, C., D-frame net collecting,; recordedBy: Koraon Wongkamhaeng; individualID: Grandidierella gilesi; individualCount: 58; sex: female; lifeStage: adult; reproductiveCondition: mature; behavior: tube builder, attached to a log at the bottom of the river; occurrenceStatus: Dominant, lives with aquatic insects; preparations: 70% alcohol; disposition: deposited at Princess Maha Chakri Sirindhorn Natural History Museum, Prince of Songkla University, Songkla, Thailand; occurrenceID: urn:catalog:PSUZC:Crus:0449; **Taxon:** taxonID: Introduced; taxonConceptID: Koraon Wongkamhaeng; scientificName: Grandidierella gilesi; kingdom: Animalia; phylum: Arthropoda; class: Malacostraca; order: Amphipoda; family: Aoridae; genus: Grandidierella ; specificEpithet: gilesi; scientificNameAuthorship: Chilton, 1921; **Location:** locationID: 14°54'06.6"N, 101°48'53.7"E; continent: Asia; waterBody: river; country: Thailand; stateProvince: Nakhon Ratchasima; county: Thailand; municipality: Sung Noen; locality: Lam Ta Khong River; verbatimElevation: 212 m; verbatimDepth: 1.5 m; verbatimCoordinates: 14°54'06.6"N, 101°48'53.7"E; georeferenceProtocol: GPS; **Identification:** identifiedBy: Koraon Wongkamhaeng; dateIdentified: 2016; **Event:** samplingProtocol: D-frame net collecting; eventDate: 07/07/2016; **Record Level:** modified: 09/05/2019; language: en; collectionCode: Crustacea; basisOfRecord: PreservedSpecimen
- **Type status:** Other material. **Occurrence:** occurrenceDetails: Asari KP, Myers AA (1982); catalogNumber: PSUZC-0448; occurrenceRemarks: loamy sand bottom, depth 1.5 m, 0 ppt; recordNumber: Boonyanusith, C., D-frame net collecting,; recordedBy: Koraon Wongkamhaeng; individualID: Grandidierella gilesi; individualCount: 18; sex: male; lifeStage: adult; reproductiveCondition: mature; behavior: tube builder, attached to a log at the bottom of the river; occurrenceStatus: Dominant, lives with aquatic insects; preparations: 70% alcohol; disposition: deposited at Princess Maha Chakri Sirindhorn Natural History Museum, Prince of Songkla University, Songkla, Thailand.; occurrenceID: urn:catalog:PSUZC:Crus:0448; **Taxon:** taxonID: Introduced; taxonConceptID: Koraon Wongkamhaeng; scientificName: Grandidierella gilesi; kingdom: Animalia; phylum: Arthropoda; class: Malacostraca; order: Amphipoda; family: Aoridae; genus: Grandidierella ; specificEpithet: gilesi; scientificNameAuthorship: Chilton, 1921; **Location:** locationID: 14°55'12.5"N, 101°51'43.2"E; continent: Asia; waterBody: river; country: Thailand; stateProvince: Nakhon Ratchasima; county: Thailand; municipality: Bung Khilek; locality: Lam Ta Khong River; verbatimElevation: 205 m; verbatimDepth: 1.5 m; verbatimCoordinates: 14°55'12.5"N, 101°51'43.2"E; georeferenceProtocol: GPS; **Identification:** identifiedBy: Koraon Wongkamhaeng; dateIdentified: 2016; **Event:** samplingProtocol: hand-collecting; eventDate: 19/09/2016; **Record Level:** modified: 09/05/2019; language: en; collectionCode: Crustacea; basisOfRecord: PreservedSpecimen
- **Type status:** Other material. **Occurrence:** occurrenceDetails: Asari KP, Myers AA (1982); catalogNumber: PSUZC-0448; occurrenceRemarks: loamy sand bottom, depth 1.5 m, 0 ppt; recordNumber: Boonyanusith, C., D-frame net collecting,; recordedBy: Koraon Wongkamhaeng; individualID: Grandidierella gilesi; individualCount: 30; sex: female; lifeStage: adult; reproductiveCondition: mature; behavior: tube builder, attached to a log at the bottom of the river; occurrenceStatus: Dominant, lives with aquatic insects; preparations: 70% alcohol; disposition: deposited at Princess Maha Chakri Sirindhorn Natural History Museum, Prince of Songkla University, Songkla, Thailand.; occurrenceID: urn:catalog:PSUZC:Crus:0448; **Taxon:** taxonID: Introduced; taxonConceptID: Koraon Wongkamhaeng; scientificName: Grandidierella gilesi; kingdom: Animalia; phylum: Arthropoda; class: Malacostraca; order: Amphipoda; family: Aoridae; genus: Grandidierella ; specificEpithet: gilesi; scientificNameAuthorship: Chilton, 1921; **Location:** locationID: 14°55'12.5"N, 101°51'43.2"E; continent: Asia; waterBody: river; country: Thailand; stateProvince: Nakhon Ratchasima; county: Thailand; municipality: Bung Khilek; locality: Lam Ta Khong River; verbatimElevation: 205 m; verbatimDepth: 1.5 m; verbatimCoordinates: 14°55'12.5"N, 101°51'43.2"E; georeferenceProtocol: GPS; **Identification:** identifiedBy: Koraon Wongkamhaeng; dateIdentified: 2016; **Event:** samplingProtocol: hand-collecting; **Record Level:** modified: 09/05/2019; language: en; collectionCode: Crustacea; basisOfRecord: PreservedSpecimen

## Impact

The amphipods have the potential to affect native fauna such as freshwater sponges via habitat modification through tube-building. In coastal polychaete aquaculture in eastern Thailand, the invasion of this amphipod species was observed and the aggressive behaviour was recorded from the farmer.

## Management

Observation and monitoring on *Grandidierella gilesi* spread are needed in other rivers in Thailand in order to prevent spreading to aquaculture areas.

## Uses

This amphipod species has been reported as a major food in the stomach contents of some economic fish species. It can be reared as live food in coastal and freshwater aquaculture.

Amphipods play many important roles in the trophodynamics link between primary producers to larger animals in higher trophic levels. This species is a filter feeder which cleans suspended particles in the water column.

## Discussion

**Figure 3.**
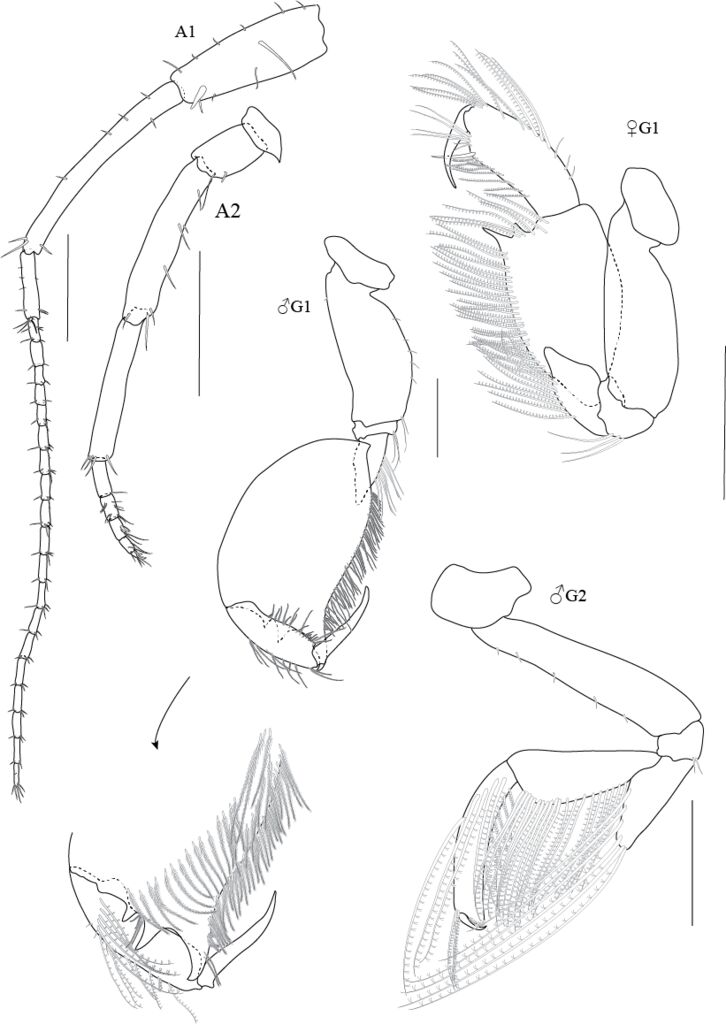
*Grandidierella gilesi* Chilton, 1921, male 6.7 mm and female 6.1 mm from Lam Ta Khong River. A1, antenna 1; A2, antenna 2; G1, gnathopod 1; G2, gnathopod 2. All scale bars represent 0.5 mm.

The *Grandidierella gilesi*, found in the present study, were similar to those described by Asari and Myers (1982) in gnathopod 2, each of which had a merus with long plumose marginal setae on the anterior margin, a carpus with two rows of long plumose setae and a propodus with long plumose marginal setae. However, one large and one small tooth on the inner face are present on the gnathopod 1 carpus of the specimens from Lam Tha Kong River, studied here (Fig. 3). Such teeth were not documented in the *G. gilesi* observed in other areas, such as the Indian Ocean (Chilton 1921, Barnard 1935, Nayar 1959, Asari and Myers 1982), the Gulf of Thailand and the South China Sea (Chilton 1925, Wongkamhaeng et al. 2016) and Australia (Myers 1981).

*Grandidierella gilesi* specimens were abundant in both study sites and present in all of the replicates. These specimens occurred in both silty clay and loamy sand and appeared to prefer deeper water. This species of amphipod is a tube builder and typically lives in a mass of detritus and debris. Some members of this species attach their tubes to sticks from trees. Along the 20 km observation area, *G. gilesi* was only found in Sung Noen and Bung Khilek. This amphipod was present all year round in Sung Noen but occurred only in the dry season (November) in Bung Khilek. It is possible that the population present in Bung Khilek was smaller and was therefore washed out during the rainy season. Several aquatic insect families can be found in the same area, including Ecnomidae, Leptoceridae, Chironomidae, Caenidae, Baetidae, Callopterygidae and Gomphidae. Freshwater clams of the genus *Cobucula* and palaemonid shrimps have also been found in this area.

A variety of reports of other non-indigenous *Grandidierella* species have discussed their introduction pathways. For example, *Grandidierella japonica* was introduced with the importation of Japanese oysters and *Crassostres gigas* (Chapman and Dorman 1975; Jourde et al. 2013) has been transported via ballast water or fouling attached to international shipping (Carlton and Eldredge 2009). Moreover, *Grandidierella bonnieroides* may also have been transported via ship fouling or ballast sediment (Jourde et al. 2013). In the case of *Grandidierella gilesi*, it has been reported in areas from brackish waters to coastal zone areas in the Indo-Pacific area (Barnard 1935, Chilton 1921, Chilton 1925, Imbach 1967, Ledoyer 1979, Lowry and Stoddart 2003, Nayar 1959, Sivaprakasam 1970, Schellenberg 1938, Wang et al. 2010) and it can also survive in mangrove forests (Bussarawich 1985). This amphipod was first reported outside of its natural range in a man-made pond at Kasetsart University in Bangkok (35 km from the sea), possibly introduced through freshwater oyster aquaculture (Wongkamhaeng et al. 2016). Barnard et al. (1991) mentioned that *G. gilesi* is a tube builder and is often associated with oyster shells; therefore, this amphipod might have entered the university pond via oyster shells.

The present study site was located approximately 200 km from the Gulf of Thailand. According to data from the Department of Fisheries, one pathway for *G. gilesi* introduction may have been related to *Litopenaeus vannamei* farms in some abandoned saltpans in None Pradu District, 50 km from the study site. *Litopenaeus vannamei* are marine shrimps. The farms in this area operate by transporting post-larvae shrimps from hatcheries in the Gulf of Thailand and directly introducing them to the farms. *Grandidierella gilesi* could have entered the hatcheries via the seawater pump or live food or could have been introduced along with the shrimp larvae. In 2011, a massive flood covered two-thirds of the country, including this area. The amphipod living in the aquaculture system may have migrated to the Lam Ta Khong River during this flood. Further sampling of the amphipod in the *L. vannamei* farm is needed. Genetic comparison between the population from the Lam Ta Khong River and other sources in Thailand might explain the origin and the process for the introduction of *G. gilesi* into this area. Another potential vector for alien amphipod introduction is ornithochory (Figuerola and Green 2002; Pandolfi et al. 2017), of which further observation is needed.

## Conclusions

The first record of the non-indigenous amphipod *Grandidierella gilesi* in the Lam Ta Khong River is presented. Previously, this Indo-Pacific amphipod was only found in estuaries and marine water areas. In Thailand, *G. gilesi* was reported in an isolated pond in Bangkok; this study constitutes another record of this species in inland waters. The male specimens from this area displayed variations on the gnathopod 2 carpus in that it is armed with one large and one small tooth on the inner face. These have not been documented in *G. gilesi*. One pathway of amphipod introduction may be related to *Litopenaeus vannamei* farms in an abandoned saltpan in the None Pradu District, 50 km from the study site. The amphipods living in this aquaculture system may have migrated to the Lam Ta Khong River during a flood that occurred in the rainy season.

## Figures and Tables

**Figure 4.**
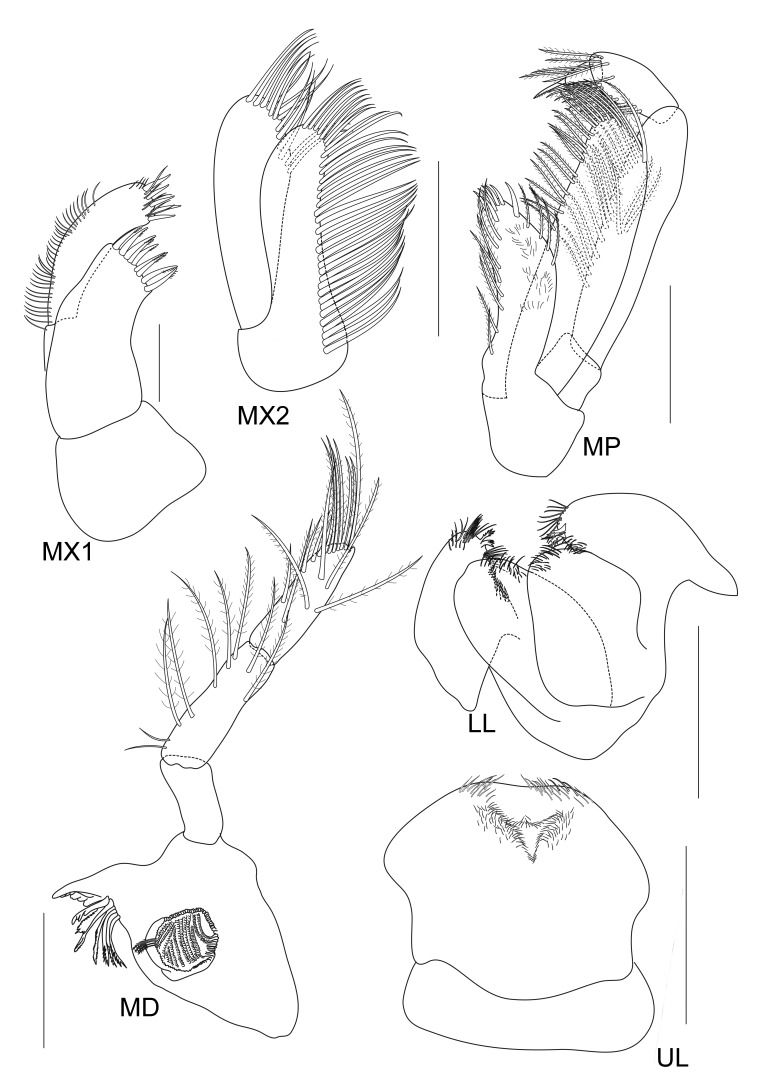
*Grandidierella gilesi* Chilton, 1921, male 6.7 mm from Lam Ta Khong River. MX1, maxilla 1; MX2, maxilla 2; MD, mandible; MP, maxilliped; LL, lower lip; UL, upper lip. All scale bars represent 0.2 mm.

**Figure 5.**
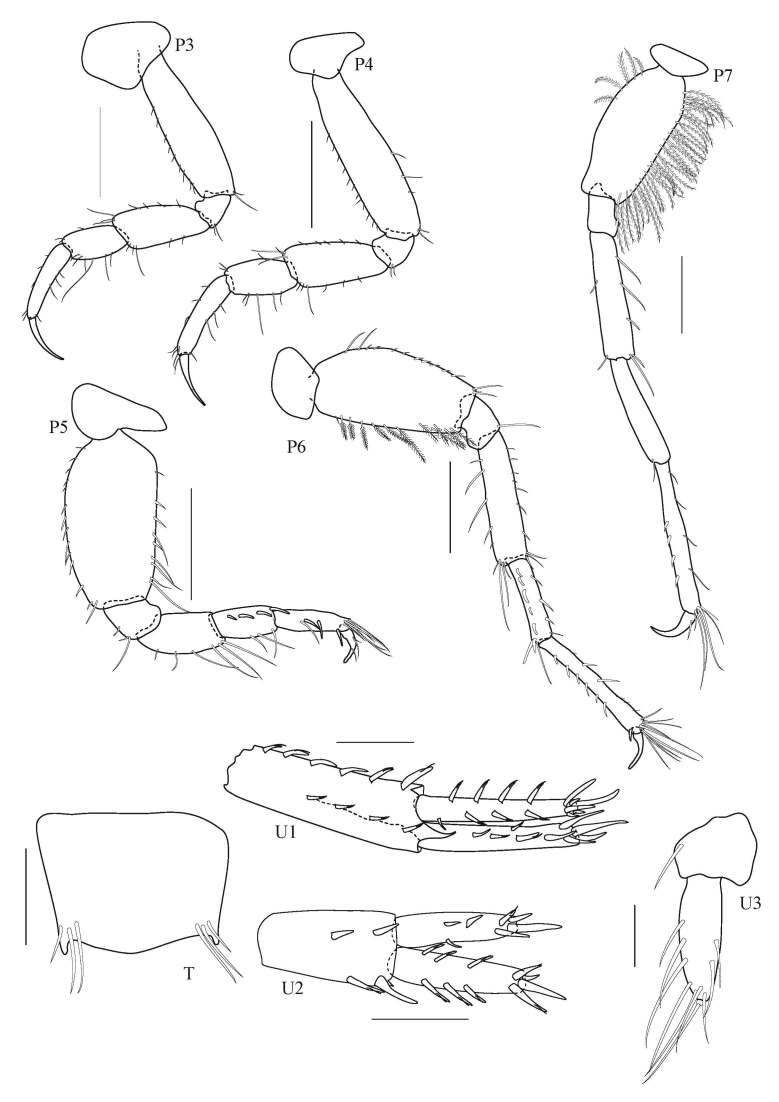
*Grandidierella gilesi* Chilton, 1921, male 6.7 mm from Lam Ta Khong River. P3, pereopod 3; P4, pereopod 4; P5, pereopod 5; P6, pereopod 6; P7, pereopod 7; U1, uropod 1; U2, uropod 2; U3, uropod 3; T, telson. All scale bars represent 0.2 mm.

## Hosting institution

Department of Zoology, Faculty of Science, Kasetsart University, Thailand, Department of Biology, Faculty of Science, Burapha University, Thailand
